# Real‐World Vortioxetine Prescription for Patients With Major Depressive Disorder in Japan: A Retrospective Cohort Study Using a Japanese Health Insurance Claims Database

**DOI:** 10.1002/npr2.70133

**Published:** 2026-06-03

**Authors:** Masaki Kato, Tatsuya Hoshino, Yayoi Kawata, Fumie Tokuda, Shinji Fujimoto

**Affiliations:** ^1^ Department of Neuropsychiatry, Faculty of Medicine Kansai Medical University Osaka Japan; ^2^ Japan Medical Office Takeda Pharmaceutical Co., Ltd Tokyo Japan

**Keywords:** antidepressant, Japan, major depressive disorder, prescriptions, vortioxetine

## Abstract

**Aim:**

Vortioxetine has been approved for the treatment of patients with major depressive disorder (MDD) in Japan since 2019; however, limited data are available on its use in clinical practice. This study aimed to identify real‐world prescribing patterns for first‐line antidepressant treatment of patients with MDD in Japan, and to understand how patient characteristics influence treatment selection and continuation.

**Methods:**

This retrospective cohort study utilized data from the JMDC claims database. Patients with MDD (aged ≥ 18 years) were included if they were newly prescribed antidepressants between November 1, 2019 and May 31, 2024. The primary outcome was the distribution of antidepressants prescribed as first‐line treatment. Secondary outcomes included evaluating treatment patterns and characteristics among patients receiving a single antidepressant as first‐line therapy.

**Results:**

The analysis population included 263 773 patients, of whom 97.4% had single‐drug prescriptions (vortioxetine [10.2%], selective serotonin reuptake inhibitors [48.3%], serotonin–norepinephrine reuptake inhibitors [15.2%], noradrenergic and specific serotonergic antidepressants [9.8%], and other antidepressants [13.9%]), and 2.6% had multiple‐drug prescriptions. Patients were less likely to be prescribed vortioxetine if they were female (odds ratio: 0.802; 95% confidence interval: 0.781–0.824) or older than 60 years (0.544; 0.498–0.594), whereas patients with somnolence (2.248; 1.152–4.385), adjustment disorders (1.488; 1.433–1.544), and nausea and vomiting (1.253; 1.194–1.315) were more likely to be prescribed vortioxetine. In the first 3 months after initiating vortioxetine treatment, 41.3% of patients continued treatment, 44.7% discontinued, and 14.0% changed treatment. Treatment change was more common in women (1.129; 1.046–1.218), and in patients with physical symptoms, whereas mental health‐related comorbidities were associated with treatment persistence.

**Conclusion:**

These findings suggest that vortioxetine is more commonly prescribed to working‐age men in Japan, and that patient characteristics and tolerability considerations contribute to prescribing and continuation patterns in clinical practice.

## Abstract 2

This study investigated the real‐world prescription patterns of antidepressants in patients with major depressive disorder in Japan. The findings suggest that vortioxetine is more commonly prescribed to working‐age men in Japan, and that patient characteristics and tolerability considerations contribute to prescribing and continuation patterns in clinical practice.

## Graphical abstract

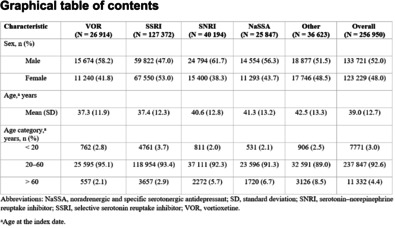

## 1 Introduction

Major depressive disorder (MDD) is a mental disorder that has a substantial impact on individuals' lives through mood, physical, and cognitive symptoms [1, 2]. The lifetime and 12‐month prevalences of MDD in Japan are 5.7% and 2.7%, respectively [3]; therefore, MDD is considered a common disease that affects many Japanese people. MDD is a complex and varied condition, with symptoms that differ between individuals. Patients with MDD also have diverse clinical backgrounds; for example, patients often have psychiatric comorbidities such as anxiety, developmental disorders, and sleep disorders [1, 2, 4, 5, 6]. Furthermore, it is common for patients with MDD to have physical comorbidities, which are managed alongside their mental health treatment [2, 7, 8].

Antidepressant therapy is one of the most promising treatment options for MDD. Treatment guidelines recommend antidepressants including selective serotonin reuptake inhibitors (SSRIs), serotonin–norepinephrine reuptake inhibitors (SNRIs), mirtazapine, and vortioxetine as first‐line treatments for patients with MDD [9, 10]. A wide range of antidepressants are approved in Japan, which each have distinct characteristics. Unlike some other countries, the Japanese medical insurance system does not categorize antidepressants into first‐ or second‐line therapies, and all approved antidepressants can be prescribed to new patients at the discretion of the physician. In practice, this means that physicians must carefully choose medications that best match each patient's symptoms and clinical background [11]. Although certain treatments may have different efficacies for specific symptoms of MDD, the overall differences in efficacy are minimal [12]. However, side effects can vary and often depend on the patient's individual characteristics. Importantly, current treatment guidelines in Japan do not provide specific recommendations for selecting antidepressants based on individual symptom profiles. As a result, there is a lack of decisive information to help in determining the most suitable medication for patients with MDD.

Vortioxetine is an antidepressant that selectively inhibits serotonin reuptake and modulates various serotonin receptors [13]. Therefore, vortioxetine modulates the release of serotonin and is also involved in the downstream release of neurotransmitters such as glutamate, dopamine, norepinephrine, acetylcholine, and histamine. Vortioxetine has been reported to improve a range of symptoms, including anxiety, cognitive dysfunction, and emotional blunting, in addition to its antidepressant effects due to its diverse serotonin receptor modulating activities [14, 15, 16]. Vortioxetine also has a long half‐life, few drug–drug interactions, and is well tolerated [10, 17, 18], which suggests that it can be prescribed to a range of patients with MDD, including those with psychiatric and physical comorbidities. In a phase 3 trial in Japan, vortioxetine improved Montgomery–Åsberg Depression Rating Scale scores and was well tolerated in patients with MDD [14]. Based on these findings, vortioxetine was approved for use for patients with MDD in Japan. However, it is unclear how well these findings translate to real‐world clinical settings, in which patients often present with more diverse and complex profiles [11]. The clinical trial data represent findings for a specific patient population, limited to those with recurrent conditions, which means that not all patient backgrounds were represented. Therefore, real‐world clinical data in Japan would be valuable for understanding the use of vortioxetine across a more diverse patient population.

Understanding the prescription patterns of antidepressants in clinical settings can offer valuable insights into optimizing treatment selection and timing for patients with MDD. In Japan, published information regarding the prescription patterns of vortioxetine is limited. Currently, there are no available data on the number of patients with MDD who have been prescribed vortioxetine or on the characteristics of patient subgroups receiving vortioxetine in Japan. However, prescription data in clinical practice have been collected since vortioxetine became available in 2019. These data reflect the real‐world perceptions and usage patterns of vortioxetine in Japan.

In this study, we used real‐world data from an insurance claims database to determine antidepressant prescription patterns and patient characteristics in Japan following the launch of vortioxetine. Our objective was to identify the prescribing patterns for patients with MDD who were newly prescribed antidepressants from November 2019, and to understand how patient characteristics such as demographics, comorbidities, and concomitant medications influenced the selection of first‐line antidepressant prescriptions and the continuation of antidepressant treatment.

## 2 Methods

### 2.1 Study Design and Patients

This retrospective cohort study utilized data from the JMDC claims database (JMDC Inc., Tokyo, Japan) from between May 1, 2019 and August 31, 2024. Japan's national health insurance system requires that all citizens enroll in one of several insurance schemes, determined by factors such as age, region, and employment status. Among these are health insurance associations that primarily cover individuals younger than 75 years old who are employed by large businesses, and their dependents [19]. The JMDC database is compiled using anonymized claims data sourced from these contracted health insurance associations, as well as from medical institutions. As of March 2025, the database included approximately 23 million individuals, offering data for analyzing prescription trends and healthcare utilization in Japan.

**FIGURE 1:**
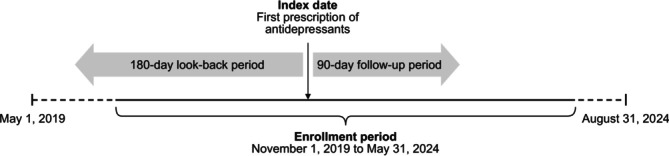
Study design.

Patients were included if they were at least 18 years old, had a diagnosis code for MDD (International Classification of Diseases 10th Revision [ICD‐10]: F32 or F33), and were newly prescribed antidepressants between November 1, 2019 and May 31, 2024 (enrollment period; Figure 1). The index date was defined as the date of the first antidepressant prescription. Eligible patients were required to have at least 180 days of data before the index prescription (look‐back period) and a minimum of 90 days of follow‐up data. New users (patients who were newly prescribed antidepressants) were defined as patients who had no record of antidepressant prescriptions during the look‐back period. Patients were excluded if they had no diagnostic code for MDD on the index date, had a diagnosis of schizophrenia (F20–F21) on the index date or during the look‐back period, were prescribed antidepressants during the look‐back period, or had insufficient follow‐up or look‐back data.

This study was a noninterventional, retrospective analysis of anonymized health insurance claims data. As such, it did not involve direct interaction with human patients or the collection of identifiable personal data. Therefore, ethical approval and informed consent were not required. The study was conducted in accordance with the ethical principles that have their origin in the Declaration of Helsinki and Good Clinical Practice. The study was registered (University Hospital Medical Information Network: UMIN000056181).

### 2.2 Outcomes

The primary outcome was the distribution of first‐line antidepressants prescribed as initial treatment for patients with MDD in Japan. Antidepressants were classified into five groups based on Anatomical Therapeutic Chemical (ATC) codes, which were: vortioxetine, SSRIs, SNRIs, noradrenergic and specific serotonergic antidepressants (NaSSAs), and other antidepressants (Table S1). Prescriptions were determined using ATC codes, and patient distribution was collected by each of the groups. In Japan, prescriptions exceeding 14 days are restricted during the first year following a new drug's listing under the National Health Insurance (NHI) system, and during this time physicians may prescribe the newly listed drug only in 14‐day increments. To account for this restriction period for vortioxetine, stratified analyses for the distribution of first‐line antidepressant prescriptions were conducted by index date (until October 31, 2020 vs. from November 1, 2020).

Secondary outcomes included evaluating treatment patterns among patients receiving a single antidepressant as first‐line therapy. For this, we analyzed treatment‐related events (treatment discontinuation, continuation, and changes) in the 3 months after initiating antidepressant therapy (follow‐up period). Treatment continuation was defined as prescriptions of a specific antidepressant with gaps of fewer than 14 days between refills. Treatment discontinuation was defined as a gap of 14 days or more between prescriptions, with the discontinuation date set as the last prescription date plus the number of days supplied. Treatment change was defined as a switch to or addition of another antidepressant. Changes within the same drug class were counted as treatment changes, but dosage increases were not. If multiple changes occurred, only the first change was counted. Additional secondary outcomes included patient characteristics (age, sex, comorbidities, and concomitant medications) for each first‐line antidepressant and treatment‐related event, and logistic regression analyses to identify factors associated with initial antidepressant selection and treatment changes. Comorbidities and concomitant medications during the look‐back period were identified using ICD‐10 and ATC codes, respectively, relevant to depression and antidepressant use based on clinical guidelines and expert consultation (Tables S2 and S3).

### 2.3 Statistical Analyses

Sample size was based on available data in the JMDC claims database. Descriptive statistics were used to summarize patient characteristics, comorbidities, concomitant medications, and treatment patterns. For continuous variables, means and standard deviations (SDs) or medians and interquartile ranges (IQRs) were calculated, depending on data distribution. Categorical variables were summarized using frequencies and percentages.

Patient demographics at the index date were obtained from the JMDC claims data. Birth dates were recorded by month; therefore, the first day of the month was used to calculate age. For comorbidities and concomitant medications, if diagnosis or prescription dates were missing, day 15 of the corresponding month was used. No formal statistical testing was performed for the analyses of patient characteristics, comorbidities, and concomitant medications by first‐line single antidepressant prescription.

To identify factors associated with treatment decisions, logistic regression analyses were conducted. We first assessed factors influencing the choice of vortioxetine as the first‐line antidepressant, with vortioxetine as the dependent variable (coded as 1 for vortioxetine and 0 for other antidepressants). We then evaluated factors associated with treatment change in the 3 months after initiating antidepressant therapy, excluding patients who discontinued treatment, with treatment change as the dependent variable (coded as 1 for treatment change and 0 for treatment continued). Explanatory variables for both analyses included age at the index date (< 20, 20–60, or > 60 years), sex, comorbidities, and concomitant medications. Multivariate logistic regression analyses were conducted using a stepwise method for variable selection to identify variables that had a statistically significant association, with a significance level of 0.05. Univariate logistic regression analyses were also performed for each explanatory variable concerning its respective dependent variable. Analyses were conducted using SAS version 9.4 (SAS Institute, Cary, NC, USA).

## 3 Results

### 3.1 Patient Disposition and Characteristics

**FIGURE 2:**
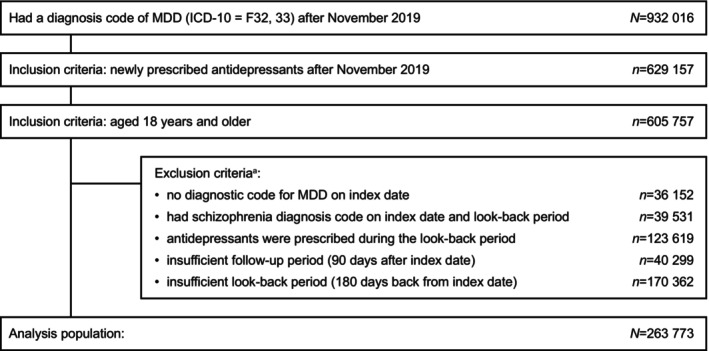
Patient flow. MDD, major depressive disorder; ICD‐10, International Classification of Diseases 10th Revision. ^a^Patients may meet more than one exclusion criterion.

Overall, 932 016 patients had a diagnosis code for MDD during the enrollment period (Figure 2). Of these, 629 157 patients were new users of antidepressants, and 263 773 met all eligibility criteria and were included in the analysis population. Patient characteristics for the analysis population are shown in Table 1; the distribution of sex was generally balanced, with a slightly higher number of men than women, and the mean (SD) age at index date was 39.0 (12.7) years.

**TABLE 1:** Patient characteristics.

Characteristic	Overall (*N* = 263 773)
Sex, *n* (%)	
Male	138 112 (52.4)
Female	125 661 (47.6)
Age,^a^ years	
Mean (SD)	39.0 (12.7)
Range	18–74
Median (IQR)	38.0 (28.0–49.0)
Age category,^a^ years, *n* (%)	
< 20	7895 (3.0)
20–60	244 287 (92.6)
> 60	11 591 (4.4)

Abbreviations: IQR, interquartile range; SD, standard deviation.

^a^ Age at the index date.

### 3.2 Distribution of First‐Line Antidepressants Prescribed for Patients With MDD

**FIGURE 3:**
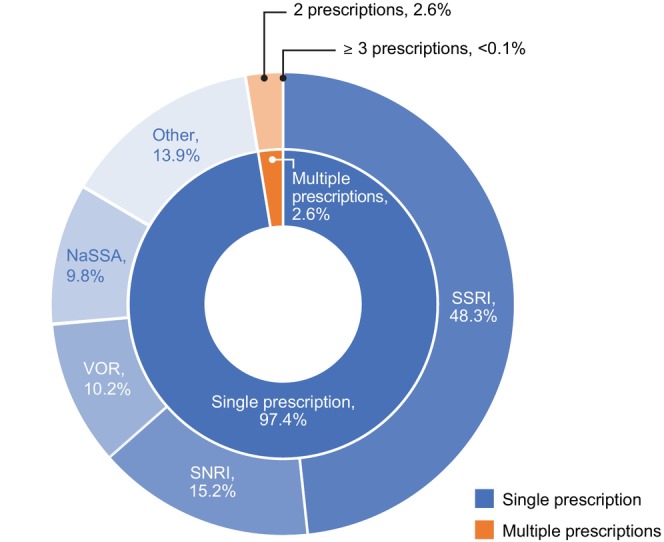
Distribution of first‐line antidepressants. NaSSA, noradrenergic and specific serotonergic antidepressant; SNRI, serotonin–norepinephrine reuptake inhibitor; SSRI, selective serotonin reuptake inhibitor; VOR, vortioxetine.

The distribution of first‐line antidepressant prescriptions is shown in Figure 3. Of the 263 773 patients in the analysis population, 97.4% were prescribed a single first‐line antidepressant, 2.6% received two antidepressants as first‐line therapy, and < 0.1% had a multiple‐drug prescription of at least three antidepressants. Overall, 10.2% had a single‐drug prescription for vortioxetine, 48.3% for an SSRI, 15.2% for an SNRI, 9.8% for an NaSSA, and 13.9% for other antidepressants.

The distribution of first‐line antidepressant prescriptions during and after the new‐drug restriction period for vortioxetine (during which there was a maximum prescription length of 14 days) is shown in Table S4. The proportion of patients who were prescribed vortioxetine was 2.7% during the restriction period (until October 31, 2020), and 12.2% after the restriction was lifted (from November 1, 2020).

### 3.3 Characteristics of Patients With MDD by First‐Line Single Antidepressant Prescription

Patient characteristics stratified by each antidepressant category are shown in Table 2. The proportion of male patients was 58.2% in the vortioxetine group, 47.0% in the SSRI group, 61.7% in the SNRI group, 56.3% in the NaSSA group, and 51.5% in the other antidepressant group. The antidepressant groups with the numerically highest proportion of patients older than 60 years old were the NaSSA group (6.7%) and those prescribed other antidepressants (8.5%).

**TABLE 2:** Patient characteristics stratified by first‐line antidepressant category for patients with single prescriptions.

Characteristic	VOR (*n* = 26 914)	SSRI (*n* = 127 372)	SNRI (*n* = 40 194)	NaSSA (*n* = 25 847)	Other (*n* = 36 623)	Overall (*n* = 256 950)
Sex, *n* (%)						
Male	15 674 (58.2)	59 822 (47.0)	24 794 (61.7)	14 554 (56.3)	18 877 (51.5)	133 721 (52.0)
Female	11 240 (41.8)	67 550 (53.0)	15 400 (38.3)	11 293 (43.7)	17 746 (48.5)	123 229 (48.0)
Age,^a^ years						
Mean (SD)	37.3 (11.9)	37.4 (12.3)	40.6 (12.8)	41.3 (13.2)	42.5 (13.3)	39.0 (12.7)
Age category,^a^ years, *n* (%)						
< 20	762 (2.8)	4761 (3.7)	811 (2.0)	531 (2.1)	906 (2.5)	7771 (3.0)
20–60	25 595 (95.1)	118 954 (93.4)	37 111 (92.3)	23 596 (91.3)	32 591 (89.0)	237 847 (92.6)
> 60	557 (2.1)	3657 (2.9)	2272 (5.7)	1720 (6.7)	3126 (8.5)	11 332 (4.4)

Abbreviations: NaSSA, noradrenergic and specific serotonergic antidepressant; SD, standard deviation; SNRI, serotonin–norepinephrine reuptake inhibitor; SSRI, selective serotonin reuptake inhibitor; VOR, vortioxetine.

^a^ Age at the index date.

Comorbidities and concomitant medications are shown in Tables S5 and S6. Overall, the most common comorbidities during the look‐back period were sleep disorders (40.5%), pain (23.3%), and anxiety disorders (22.0%). In the vortioxetine group, there was a numerically higher proportion of patients with adjustment disorders (14.9%) than the overall rate (10.5%). The most prescribed concomitant medications during the look‐back period, both overall and in the vortioxetine group, were: antacids, bowel regulators, and ulcer medications (overall: 32.7%; vortioxetine: 28.6%); analgesics (overall: 28.5%; vortioxetine: 25.5%); anti‐inflammatory and anti‐rheumatic products (overall: 27.4%; vortioxetine: 24.9%); anxiolytics (overall: 25.2%; vortioxetine: 23.1%); and hypnotics and sedatives (overall: 24.2%; vortioxetine: 22.3%).

### 3.4 Treatment Patterns Among Patients With MDD Prescribed a Single First‐Line Antidepressant

Treatment patterns during the first 3 months following initial prescription are shown in Table 3. During this period, 37.1% of patients continued treatment, 49.3% discontinued, and 13.7% experienced a treatment change (8.9% switched to a different drug and 4.8% had another drug added). In the vortioxetine group, 41.3% of patients continued treatment, 44.7% discontinued, and 14.0% underwent a treatment change (8.9% switched medications and 5.1% had an additional drug introduced).

**TABLE 3:** Treatment patterns during the 3 months after initial prescription for patients with single prescriptions.

	VOR (*n* = 26 914)	SSRI (*n* = 127 372)	SNRI (*n* = 40 194)	NaSSA (*n* = 25 847)	Other (*n* = 36 623)	Overall (*N* = 256 950)
Patients who continued treatment, *n* (%)	11 111 (41.3)	52 067 (40.9)	15 125 (37.6)	7773 (30.1)	9162 (25.0)	95 238 (37.1)
Patients who discontinued treatment, *n* (%)	12 029 (44.7)	58 835 (46.2)	19 525 (48.6)	13 344 (51.6)	22 888 (62.5)	126 621 (49.3)
Patients who had a treatment change, *n* (%)	3774 (14.0)	16 470 (12.9)	5544 (13.8)	4730 (18.3)	4573 (12.5)	35 091 (13.7)
Patients who switched treatment, *n* (%)^a^	2399 (8.9)	10 959 (8.6)	3554 (8.8)	3156 (12.2)	2732 (7.5)	22 800 (8.9)
Switched to VOR	0 (0.0)	1547 (14.1)	566 (15.9)	488 (15.5)	198 (7.2)	2799 (12.3)
Switched to SSRI	1096 (45.7)	4427 (40.4)	1473 (41.4)	1433 (45.4)	838 (30.7)	9267 (40.6)
Switched to SNRI	755 (31.5)	2838 (25.9)	556 (15.6)	741 (23.5)	467 (17.1)	5357 (23.5)
Switched to NaSSA	398 (16.6)	1410 (12.9)	516 (14.5)	0 (0.0)	675 (24.7)	2999 (13.2)
Switched to other antidepressant	191 (8.0)	904 (8.2)	493 (13.9)	550 (17.4)	601 (22.0)	2739 (12.0)
Patients who had a treatment addition, *n* (%)^b^	1375 (5.1)	5511 (4.3)	1990 (5.0)	1574 (6.1)	1841 (5.0)	12 291 (4.8)
Added VOR	0 (0.0)	258 (4.7)	133 (6.7)	179 (11.4)	203 (11.0)	773 (6.3)
Added SSRI	176 (12.8)	596 (10.8)	286 (14.4)	506 (32.1)	793 (43.1)	2357 (19.2)
Added SNRI	363 (26.4)	1243 (22.6)	72 (3.6)	681 (43.3)	385 (20.9)	2744 (22.3)
Added NaSSA	341 (24.8)	1089 (19.8)	693 (34.8)	0 (0.0)	193 (10.5)	2316 (18.8)
Added other antidepressant	497 (36.1)	2338 (42.4)	808 (40.6)	214 (13.6)	275 (14.9)	4132 (33.6)

Abbreviations: NaSSA, noradrenergic and specific serotonergic antidepressant; SNRI, serotonin–norepinephrine reuptake inhibitor; SSRI, selective serotonin reuptake inhibitor; VOR, vortioxetine.

^a^ Percentages for patients who switched to specific antidepressant groups are based on the total number in this row. Each patient contributes only one switch event (only the first treatment change was counted); however, a single switch may involve multiple antidepressant classes, so patients may appear in more than one category.

^b^ Percentages for patients who added specific antidepressant groups are based on the total numbers in this row. Each patient contributes only one addition event (only the first treatment change was counted); however, a single addition may involve multiple antidepressant classes, so patients may appear in more than one category.

### 3.5 Factors Influencing Antidepressant Selection

Results of the logistic regression analyses for factors associated with the selection of vortioxetine as a first‐line antidepressant are shown in Table 4 and Table S7. Logistic regression identified that women were less likely to be prescribed vortioxetine compared with men (odds ratio [OR]: 0.802; 95% confidence interval [CI]: 0.781–0.824). Patients older than 60 years also had lower odds of receiving vortioxetine (0.544; 0.498–0.594) than those aged 20–60 years. Patients with certain comorbidities during the look‐back period were more likely to be prescribed vortioxetine, including somnolence (2.248; 1.152–4.385), adjustment disorders (1.488; 1.433–1.544), nausea and vomiting (1.253; 1.194–1.315), bipolar disorder (1.192; 1.100–1.292), and sleep disorders (1.184; 1.148–1.222). In contrast, lower odds of vortioxetine prescription were observed in patients with obsessive‐compulsive disorder (0.450; 0.373–0.542), Parkinson's disease (0.666; 0.494–0.896), and substance use disorders other than alcohol (0.698; 0.512–0.951). For concomitant medications during the look‐back period, antipsychotics (1.052; 1.005–1.100) and anti‐inflammatory and anti‐rheumatic products (1.051; 1.016–1.087) were positively associated with vortioxetine use. Conversely, antiemetics and antinauseants (0.465; 0.347–0.623), antiepileptic drugs (0.812; 0.737–0.895), and hypnotics and sedatives (0.855; 0.823–0.889) were associated with lower odds of vortioxetine prescription.

**TABLE 4:** Logistic regression analysis for factors associated with the choice of vortioxetine as first‐line antidepressant.

Explanatory variable	Odds ratio (95% CI)^a^
Sex (reference: male)	
Female	0.802 (0.781–0.824)
Age^b^ (reference: 20–60 years)	
< 20 years	0.904 (0.838–0.976)
> 60 years	0.544 (0.498–0.594)
Comorbidities (reference: no)	
Somnolence	2.248 (1.152–4.385)
Adjustment disorders	1.488 (1.433–1.544)
Nausea and vomiting	1.253 (1.194–1.315)
Bipolar disorder	1.192 (1.100–1.292)
Sleep disorders	1.184 (1.148–1.222)
Hyperactivity disorder	1.157 (1.063–1.259)
Anxiety disorders	0.949 (0.917–0.981)
Cancer	0.945 (0.900–0.993)
Somatoform disorders	0.899 (0.852–0.949)
Hypertensive disorders	0.892 (0.853–0.933)
Pain	0.884 (0.852–0.917)
Constipation	0.880 (0.832–0.930)
Epilepsy	0.867 (0.768–0.978)
Headaches	0.832 (0.799–0.867)
Other noninfectious gastroenteritis and noninfectious colitis	0.810 (0.704–0.932)
Alcohol use disorders	0.740 (0.602–0.909)
Other substance use disorders	0.698 (0.512–0.951)
Premenstrual syndrome	0.694 (0.576–0.836)
Parkinson's disease	0.666 (0.494–0.896)
Obsessive‐compulsive disorder	0.450 (0.373–0.542)
Concomitant medications (reference: no)	
Antipsychotics	1.052 (1.005–1.100)
Anti‐inflammatory and anti‐rheumatic products	1.051 (1.016–1.087)
Anxiolytics	0.953 (0.920–0.987)
Drugs for functional gastrointestinal disorders	0.944 (0.907–0.983)
Anesthetics	0.909 (0.867–0.953)
Topical anti‐rheumatic drugs	0.902 (0.862–0.943)
Systemic corticosteroids	0.894 (0.852–0.937)
Hypnotics and sedatives	0.855 (0.823–0.889)
Antiepileptic drugs	0.812 (0.737–0.895)
Antiemetics and antinauseants	0.465 (0.347–0.623)

Abbreviation: CI, confidence interval.

^a^ All factors were statistically significant at *p* < 0.05 in stepwise multivariate logistic regression.

^b^ Age at the index date.

### 3.6 Factors Influencing Antidepressant Treatment Change

Results of the logistic regression analyses for factors associated with treatment change in patients prescribed vortioxetine as a first‐line antidepressant are shown in Table 5 and Table S8. In the logistic regression, women had higher odds of treatment change than men (OR: 1.129; 95% CI: 1.046–1.218). For comorbidities recorded during the look‐back period, the strongest associations with treatment change were observed for nausea and vomiting (1.197; 1.056–1.357), sleep disorders (1.155; 1.069–1.248), and headaches (1.133; 1.011–1.270). In contrast, somatoform disorders (0.835; 0.716–0.975), adjustment disorders (0.858; 0.771–0.955), and anxiety disorders (0.901; 0.821–0.989) were associated with lower odds of treatment change in patients prescribed vortioxetine. For concomitant medications during the look‐back period, intestinal disease preparations (1.347; 1.210–1.501), antipsychotics (1.175; 1.049–1.317), and anti‐inflammatory and anti‐rheumatic products (1.132; 1.032–1.241) were most strongly associated with increased likelihood of treatment change. Conversely, renin–angiotensin system agents (0.821; 0.699–0.964) and topical anti‐rheumatic drugs (0.873; 0.768–0.992) were associated with lower odds of treatment change in patients prescribed vortioxetine.

**TABLE 5:** Logistic regression analysis for factors associated with treatment change in patients prescribed vortioxetine.

Explanatory variable	Odds ratio (95% CI)^a^
Sex (reference: male)	
Female	1.129 (1.046–1.218)
Comorbidities (reference: no)	
Nausea and vomiting	1.197 (1.056–1.357)
Sleep disorders	1.155 (1.069–1.248)
Headaches	1.133 (1.011–1.270)
Anxiety disorders	0.901 (0.821–0.989)
Adjustment disorders	0.858 (0.771–0.955)
Somatoform disorders	0.835 (0.716–0.975)
Concomitant medications (reference: no)	
Intestinal disease preparations	1.347 (1.210–1.501)
Antipsychotics	1.175 (1.049–1.317)
Anti‐inflammatory and anti‐rheumatic products	1.132 (1.032–1.241)
Topical anti‐rheumatic drugs	0.873 (0.768–0.992)
Renin–angiotensin system agents	0.821 (0.699–0.964)

Abbreviation: CI, confidence interval.

^a^ All factors were statistically significant at *p* < 0.05 in stepwise multivariate logistic regression.

The results of logistic regression analyses examining factors associated with treatment changes in patients prescribed any single first‐line antidepressant (overall group), SSRIs, SNRIs, NaSSAs, or other antidepressants are presented in Table S9. In the overall group, compared with patients aged 20–60 years, the OR (95% CI) was 1.091 (1.012–1.176) for patients aged younger than 20 years, and 0.893 (0.834–0.957) for patients aged older than 60 years. Regarding comorbidities during the look‐back period, the highest ORs were observed for Parkinson's disease (1.259; 1.021–1.553) and sleep disorders (1.189; 1.158–1.220). The lowest ORs were seen for dementia (0.363; 0.194–0.681), autism (0.447; 0.229–0.872), and obsessive‐compulsive disorder (0.784; 0.693–0.888). For concomitant medications used during the look‐back period, the highest ORs were for antipsychotics (1.175; 1.129–1.223) and intestinal disease preparations (1.150; 1.107–1.195). The lowest ORs were for antiemetics and antinauseants (0.773; 0.630–0.947), and psychostimulants, attention‐deficit/hyperactivity disorder (ADHD) medications, and psychotropics (0.784; 0.680–0.904). Several factors, such as sleep disorders, were consistent across most antidepressant categories, whereas some were specific to vortioxetine (such as nausea and vomiting).

## 4 Discussion

In this retrospective cohort study, vortioxetine was prescribed as a first‐line antidepressant in 10.2% of prescriptions for patients with MDD in Japan. This suggests that clinicians have recognized its characteristics in clinical practice and that vortioxetine has become one of the major first‐line treatment options for MDD since it became available in Japan in 2019. In the first 3 months after initiating vortioxetine treatment, a similar continuation rate was observed compared with SSRIs, and a modest but numerical trend toward higher continuation rates was observed with vortioxetine compared with any other type of antidepressants, suggesting that vortioxetine may be perceived as a well‐tolerated and user‐friendly option, despite being a relatively new antidepressant. Additionally, demographic characteristics, comorbidities, and concomitant medications associated with the selection of vortioxetine as a first‐line antidepressant, and rates of subsequent changes from vortioxetine, offer insights into the use of this antidepressant in Japan.

The particular comorbidities and concomitant medications evaluated in this study were prespecified based on clinical relevance according to treatment guidelines [9, 10], product labelling and expert guidance. Among patients treated with any first‐line antidepressant, changes in treatment were associated with several comorbidities, especially neurological, psychiatric and neurodevelopmental disorders, as well as different medication classes. The comorbidities and concomitant medications associated with treatment changes differed depending on the class of antidepressant, though sleep disorders and intestinal disease preparations were consistently observed across most classes. These findings are broadly consistent with Japanese expert consensus recommendations, which support flexible pharmacological approaches to depression, tailored to individual patient needs and medication profiles [11].

Within this context, our results suggest that, in Japan, vortioxetine is more frequently prescribed for men than women, and for patients aged 20–60 years, compared with patients aged younger than 20 years or older than 60 years. This prescribing pattern aligns with the demographic profile of Japan's working population, which suggests that vortioxetine is considered a suitable option for this group. A previous study indicated that vortioxetine is unlikely to cause somnolence or insomnia [18], which are important considerations for maintaining social engagement, employment, and daily activities. Consistently, our analysis indicated that vortioxetine was more likely to be selected when sleep disorders, particularly somnolence, were present as comorbidities, suggesting that vortioxetine is perceived among antidepressants as having minimal sedative effects. Moreover, as work productivity depends not only on limited somnolence but also on cognitive function [20], our finding that vortioxetine may be a favorable option for the working population is supported by previous studies showing that vortioxetine treatment is associated with improved cognitive function scale scores in patients with MDD [21]. This is also aligned with the Canadian Network for Mood and Anxiety Treatments guidelines, which recommend vortioxetine as a first‐line option for cognitive dysfunction [10]. In Japan, prescriptions exceeding 14 days are restricted during the first year following a new drug's price listing under the NHI. The comparison of prescription patterns before and after this restriction period for vortioxetine showed an increase in vortioxetine prescriptions 1 year after price listing. This shift corresponded with a decline in prescriptions for other antidepressants, most notably SNRIs, which suggests that vortioxetine may be recognized as an alternative option to SNRIs. Given that SNRIs are often selected for their positive effects on cognition [15] and motivation [11], the trend in prescription patterns observed in our study could further indicate that vortioxetine is being prescribed to the working population to support work productivity.

Comorbidities commonly observed in patients prescribed vortioxetine as a first‐line antidepressant included sleep disorders and anxiety disorders, both of which are generally associated with depression, and are also prevalent among patients receiving other antidepressants. Adjustment disorders were also relatively common in the group prescribed vortioxetine. Notably, when adjustment disorders were present as comorbidities, vortioxetine was less likely to be changed for another treatment. This suggests that vortioxetine may be suitable for patients with depression who have adjustment disorders influenced by environmental stressors. Given that the working environment in Japan has been linked to the onset of adjustment disorders [22, 23], the association between adjustment disorders and the selection of vortioxetine aligns with the possibility that this antidepressant is frequently prescribed to the working population.

Another notable finding regarding vortioxetine prescription patterns was the association with somatoform disorder comorbidities. Although somatoform disorders were associated with a lower likelihood of vortioxetine being selected as a first‐line antidepressant, treatment change was less likely once vortioxetine was prescribed. In contrast, somatoform disorders did not show similar trends with other antidepressants. Combined with the findings that anxiety disorders and adjustment disorders were also associated with lower rates of treatment change, these results suggest that patients with mental health‐related comorbidities (such as mood and anxiety‐related comorbidities) may be more likely to continue vortioxetine treatment, indicating that these patients may be experiencing symptom relief and clinical benefit. In contrast, physical symptoms such as gastrointestinal or neurological symptoms were associated with higher rates of treatment change from vortioxetine. Interestingly, vortioxetine was more frequently prescribed to patients with nausea or vomiting than other antidepressants. Both nausea and vomiting have been reported as side effects of vortioxetine in clinical trials [14, 18]; however, a meta‐analysis concluded that vortioxetine treatment was significantly associated with nausea but not vomiting [24]. It is possible that Japanese clinicians may have the impression that even if there are gastrointestinal side effects with vortioxetine treatment, they are relatively mild in actual clinical practice. If these symptoms do develop, a change in treatment may be necessary.

This study has several limitations. The analysis was restricted to individuals enrolled in employment‐based health insurance, which excludes those aged 75 years and older, retirees, and non‐working individuals. Therefore, selection bias may limit generalizability, warranting further studies using alternative databases comprising elderly, retired, and non‐working populations. Due to the inherent limitations of claims data, it was not possible to fully distinguish between bipolar disorder and MDD in this study. Consequently, the observed treatment patterns may also reflect those of patients with bipolar disorder. Additionally, claims data do not confirm actual prescribed drug use or capture disease severity and duration, which are factors that may influence prescribing decisions. Next, no adjustment for multiple testing was applied; therefore, the results from regression analyses should be interpreted with caution. Finally, long‐term treatment patterns and outcomes were not assessed.

## 5 Conclusion

This study investigated the real‐world prescription patterns of antidepressants in patients with MDD in Japan and found that vortioxetine was prescribed most frequently to patients who were male and between 20 and 60 years of age. Vortioxetine was often prescribed to patients who had sleep disorders, adjustment disorders, and somnolence before their diagnosis of MDD. Continuation of vortioxetine treatment was more common in patients with mental health‐related comorbidities, whereas treatment changes were more likely in patients with physical symptoms such as nausea, vomiting, and headaches. These findings suggest that Japanese clinicians perceive vortioxetine as a suitable option for working‐age patients with cognitive or sleep‐related symptoms, and that its tolerability influences prescribing and continuation decisions. Further research is required to explore long‐term real‐world outcomes of vortioxetine in diverse clinical settings.

## Author Contributions

Masaki Kato: study design, data interpretation. Tatsuya Hoshino: study conceptualization, study design, data interpretation. Yayoi Kawata: study design, data interpretation. Fumie Tokuda: study design, formal analysis. Shinji Fujimoto: formal analysis, data interpretation. All authors reviewed the manuscript for important intellectual content and approved the final version.

## Funding

This database study was funded by Takeda Pharmaceutical Co. Ltd.

## Ethics Statement

This study was a noninterventional, retrospective cohort study using anonymized health insurance claims data. As such, it did not involve direct interaction with human patients or the collection of identifiable personal data. Therefore, ethical approval was not required. The study was conducted in accordance with ethical principles that have their origin in the Declaration of Helsinki. The study was registered (University Hospital Medical Information Network: UMIN000056181). This study was conducted in compliance with the Japanese Ministerial Ordinance on Good Post‐Marketing Study Practice. Institutional Review Board approval was not required according to the Good Post‐Marketing Study Practice Ordinance.

## Consent

No direct patient contact or primary collection of individual human data occurred in this study. JMDC claims data are anonymized and fully deidentified, therefore, patient informed consent was not required.

## Conflicts of Interest

Masaki Kato has received grant funding from the Japan Society for the Promotion of Science, Japan Research Foundation for Clinical Pharmacology, and SENSHIN Medical Research Foundation; consulting fees from Lundbeck Japan K.K., Otsuka Pharmaceutical Co. Ltd., Shionogi & Co. Ltd., Sumitomo Pharma Co. Ltd., and Takeda Pharmaceutical Co. Ltd.; payment or honoraria for lectures, presentations, speakers' bureaus, manuscript writing, or educational events from Eisai Co. Ltd., Eli Lilly Japan K.K., Janssen Pharmaceutical K.K., Kyowa Pharmaceutical Industry Co. Ltd., Lundbeck Japan K.K., Meiji Seika Pharma Co. Ltd., Mitsubishi Tanabe Pharma Corporation, MSD K.K., Ono Pharmaceutical Co. Ltd., Otsuka Pharmaceutical Co. Ltd., Pfizer Japan Inc., Shionogi & Co. Ltd., Sumitomo Pharma Co. Ltd., Takeda Pharmaceutical Co. Ltd., and Viatris Inc. Tatsuya Hoshino, Yayoi Kawata, Fumie Tokuda, and Shinji Fujimoto are employees of Takeda Pharmaceutical Co. Ltd.

## Supporting information


**Table S1:** Target antidepressants in this study.
**Table S2:** Comorbidities during the look‐back period.
**Table S3:** Concomitant medications during the look‐back period.
**Table S4:** Distribution of first‐line drugs among patients for the stratified analysis.
**Table S5:** Comorbidities during the look‐back period among patients with single prescriptions.
**Table S6:** Concomitant medications during the look‐back period among patients with single prescriptions.
**Table S7:** Univariate regression analysis for the factors influencing the choice of vortioxetine as a first‐line drug.
**Table S8:** Univariate regression analysis for factors influencing treatment change of vortioxetine.
**Table S9:** Logistic regression analysis for factors influencing the treatment change of antidepressants.

## Data Availability

The health insurance data used in this study are available from JMDC Inc. Access to these data is limited and not publicly available because they were used under a license for the current study.
