# Supplementary material for: Real‐World Vortioxetine Prescription for Patients With Major Depressive Disorder in Japan: A Retrospective Cohort Study Using a Japanese Health Insurance Claims Database
Source: Neuropsychopharmacol Rep. 2026 Jun 3;46(2):e70133. doi: 10.1002/npr2.70133 (PMC13240034; doi:10.1002/npr2.70133)
Supplement: Supplementary file 1 — Table S1: Target antidepressants in this study. Table S2: Comorbidities during the look‐back period. Table S3: Concomitant medications during the look‐back period. Table S4: Distribution of first‐line drugs among patients for the stratified analysis. Table S5: Comorbidities during the look‐back period among patients with single prescriptions. Table S6: Concomitant medications during the look‐back period among patients with single prescriptions. Table S7: Univariate regression analysis for the factors influencing the choice of vortioxetine as a first‐line drug. Table S8: Univariate regression analysis for factors influencing treatment change of vortioxetine. Table S9: Logistic regression analysis for factors influencing the treatment change of antidepressants. [file NPR2-46-e70133-s001.docx]

**Real-world Vortioxetine Prescription for Patients With Major Depressive Disorder in Japan: A Retrospective Cohort Study Using a Japanese Health Insurance Claims Database**

Masaki Kato^1^ | Tatsuya Hoshino^2^ | Yayoi Kawata^2^ | Fumie Tokuda^2^ | Shinji Fujimoto^2^

^1^Department of Neuropsychiatry, Faculty of Medicine, Kansai Medical University, Osaka, Japan

^2^Japan Medical Office, Takeda Pharmaceutical Co., Ltd, Tokyo, Japan

**Supporting information**

**TABLE S1 |** Target antidepressants in this study.

| **Group** | **ATC code** | **Subcategory** |
| --- | --- | --- |
| **VOR** | N06A9 | Vortioxetine Hydrobromide |
| **SSRI** | N06A4 | Fluvoxamine Maleate / Paroxetine hydrochloride hydrate (Paroxetine Hydrochloride) / Sertraline Hydrochloride / Escitalopram Oxalate |
| **SNRI** | N06A5 | Milnacipran Hydrochloride / Duloxetine Hydrochloride / Venlafaxine Hydrochloride |
| **NaSSA** | N06A9 | Mirtazapine |
| **Other** | N06A9 | Amitriptyline Hydrochloride / Amoxapine / Imipramine Hydrochloride / Clomipramine Hydrochloride / Setiptiline Maleate / Trazodone Hydrochloride / Nortriptyline Hydrochloride / Maprotiline Hydrochloride / Mianserin Hydrochloride / Lofepramine Hydrochloride / Dothiepin hydrochloride (Dosulepin Hydrochloride) / Trimipramine Maleate |

Abbreviations: ATC, Anatomical Therapeutic Chemical; NaSSA, noradrenergic and specific serotonergic antidepressant; SNRI, serotonin–norepinephrine reuptake inhibitor; SSRI, selective serotonin reuptake inhibitor; VOR, vortioxetine.

**TABLE S2 |** Comorbidities^a^ during the look-back period.

| **Disease** | **ICD-10 category/subcategory/**  **chapter** | **Description** |
| --- | --- | --- |
| Alcohol use disorders | F10 | Mental and behavioural disorders due to use of alcohol |
| Other substance use disorders | F11 | Mental and behavioural disorders due to use of opioids |
|  | F12 | Mental and behavioural disorders due to use of cannabinoids |
|  | F13 | Mental and behavioural disorders due to use of sedatives or hypnotics |
|  | F15 | Mental and behavioural disorders due to use of other stimulants, including caffeine |
|  | F16 | Mental and behavioural disorders due to use of hallucinogens |
|  | F17 | Mental and behavioural disorders due to use of tobacco |
|  | F18 | Mental and behavioural disorders due to use of volatile solvents |
|  | F19 | Mental and behavioural disorders due to multiple drug use and use of other psychoactive substances |
| Bipolar disorder | F31 | Bipolar affective disorder |
| Depression | F320 | Mild depressive episode |
|  | F321 | Moderate depressive episode |
|  | F322 | Severe depressive episode without psychotic symptoms |
|  | F323 | Severe depressive episode with psychotic symptoms |
|  | F328 | Other depressive episodes |
|  | F329 | Depressive episode, unspecified |
|  | F330 | Recurrent depressive disorder, current episode mild |
|  | F331 | Recurrent depressive disorder, current episode moderate |
|  | F332 | Recurrent depressive disorder, current episode severe without psychotic symptoms |
|  | F333 | Recurrent depressive disorder, current episode severe with psychotic symptoms |
|  | F334 | Recurrent depressive disorder, currently in remission |
|  | F338 | Other recurrent depressive disorders |
|  | F339 | Recurrent depressive disorder, unspecified |
| Anxiety disorders | F40 | Phobic anxiety disorders |
|  | F401 | Social phobias |
|  | F410 | Panic disorder [episodic paroxysmal anxiety] |
|  | F411 | Generalized anxiety disorder |
|  | F400 | Agoraphobia |
|  | F402 | Specific (isolated) phobias |
|  | F409 | Phobic anxiety disorder, unspecified |
|  | F41 | Other anxiety disorders |
|  | F412 | Mixed anxiety and depressive disorder |
|  | F418 | Other specified anxiety disorders |
|  | F419 | Anxiety disorder, unspecified |
| Obsessive-compulsive disorder | F42 | Obsessive-compulsive disorder |
| PTSD | F431 | Post-traumatic stress disorder |
| Adjustment disorders | F432 | Adjustment disorders |
| Other stress reactions | F430 | Acute stress reaction |
|  | F439 | Reaction to severe stress, unspecified |
| Dissociative disorders | F44 | Dissociative [conversion] disorders |
| Somatoform disorders | F45 | Somatoform disorders |
| Sleep disorders | F51 | Nonorganic sleep disorders |
|  | G47 | Sleep disorders |
| Personality disorders | F60 | Specific personality disorders |
| Autism | F840 | Childhood autism |
|  | F841 | Atypical autism |
| Hyperactivity disorder | F90 | Hyperkinetic disorders |
| Dementia | F01 | Vascular dementia |
|  | G300 | Alzheimer disease with early onset |
|  | G301 | Alzheimer disease with late onset |
|  | G308 | Other Alzheimer disease |
|  | G309 | Alzheimer disease, unspecified |
|  | 8845840^b^ | Dementia with Lewy bodies |
| Parkinson’s disease | G20 | Parkinson’s disease |
| Epilepsy | G40 | Epilepsy |
|  | G41 | Status epilepticus |
| Headaches | G43 | Migraine |
|  | G44 | Other headache syndromes |
|  | R51 | Headache |
| Autonomic nervous system disorders | G90 | Disorders of autonomic nervous system |
| Pain |  | All codes including “pain” except for G43, G44, and R51 |
| Cancer | C00-D48 | Neoplasms |
| Diabetes mellitus | E10-E14 | Diabetes mellitus |
| Hypertensive disorders | I10-I15 | Hypertensive diseases |
| Hyperlipidemia | E78 | Disorders of lipoprotein metabolism and other lipidaemias |
| Other noninfectious gastroenteritis and noninfectious colitis | K52 | Other noninfective gastroenteritis and colitis |
| Irritable bowel syndrome | K58 | Irritable bowel syndrome |
| Nausea and vomiting | R11 | Nausea and vomiting |
| Hypothyroidism | E03 | Other hypothyroidism |
| Constipation | K590 | Constipation |
| Diarrhea | K591 | Functional diarrhoea |
| Somnolence | R400 | Somnolence |
| Premenstrual syndrome | N943 | Premenstrual tension syndrome |

Abbreviations: ICD-10, International Classification of Diseases 10th Revision; PTSD, post-traumatic stress disorder.

^a^Comorbidity was operationally defined as any diagnosis recorded during the look-back period.
^b^Standard disease code used in claims data.

**TABLE S3 |** Concomitant medications during the look-back period.

| **Medication** | **ATC code** | **Description** |
| --- | --- | --- |
| Anesthetics | N01 | Anesthetics |
| Analgesics | N02 | Analgesics |
| Antiepileptic drugs | N03 | Antiepileptics |
| Antiparkinsonian | N04 | Anti-parkinson drugs |
| Antipsychotics | N05A | Antipsychotics |
|  | N05AL01 | Sulpiride |
| Anxiolytics | N05B | Anxiolytics |
| Hypnotics and sedatives | N05C | Hypnotics and sedatives |
|  | N05CD | Benzodiazepine derivatives |
|  | N05CF | Benzodiazepine related drugs |
|  | N05CH | Melatonin receptor agonists |
|  | N05CJ | Orexin receptor antagonists |
|  | N05CA | Barbiturates, plain |
|  | N05CB | Barbiturates, combinations |
|  | N05CC | Aldehydes and derivatives |
|  | N05CE | Piperidinedione derivatives |
|  | N05CM | Other hypnotics and sedatives |
|  | N05CX | Hypnotics and sedatives in combination, excl. barbiturates |
| Antidepressants | N06A | Antidepressants |
| Psychostimulants, ADHD medications and psychotropics | N06B | Psychostimulants, agents used for ADHD and nootropics |
| Antidementia drugs | N06D | Anti-dementia drugs |
| Other nervous system medications | N07 | Other nervous system drugs |
| Antacids, bowel regulators, and ulcer medications | A02 | Drugs for acid related disorders |
| Drugs for functional gastrointestinal disorders | A03 | Drugs for functional gastrointestinal disorders |
| Antiemetics and antinauseants | A04 | Antiemetics and antinauseants |
| Laxatives | A06 | Drugs for constipation |
| Intestinal disease preparations | A07 | Antidiarrheals, intestinal antiinflammatory/antiinfective agents |
| Diabetes medications | A10 | Drugs used in diabetes |
| Calcium channel blockers | C08 | Calcium channel blockers |
| Renin–angiotensin system agents | C09 | Agents acting on the renin-angiotensin system |
| Lipid control/ atherosclerosis | C10 | Lipid modifying agents |
| Anti-inflammatory and anti-rheumatic products | M01 | Antiinflammatory and antirheumatic products |
| Topical anti-rheumatic drugs | M02 | Topical products for joint and muscular pain |
| Systemic corticosteroids | H02A | Corticosteroids for systemic use, plain |
|  | H02B | Corticosteroids for systemic use, combinations |
| Thyroid therapeutics | H03A | Thyroid preparations |
|  | H03B | Antithyroid preparations |
| Interferon | L03AB | Interferons |

Abbreviations: ADHD, attention-deficit/hyperactivity disorder; ATC, Anatomical Therapeutic Chemical Classification System.

**TABLE S4 |** Distribution of first-line drugs among patients for the stratified analysis.

| **Variable** | **Prescriptions up to October 31, 2020**  **(n= 54 988)** | **Prescriptions from November 1, 2020**  **(n = 208 785)** |
| --- | --- | --- |
| Single prescription | 53 407 (97.1) | 203 543 (97.5) |
| VOR | 1480 (2.7) | 25 434 (12.2) |
| SSRI | 27 824 (50.6) | 99 548 (47.7) |
| SNRI | 10 180 (18.5) | 30 014 (14.4) |
| NaSSA | 5710 (10.4) | 20 137 (9.6) |
| Other | 8213 (14.9) | 28 410 (13.6) |
| 2 drugs | 1568 (2.9) | 5205 (2.5) |
| ≥ 3 drugs | 13 (< 0.1) | 37 (< 0.1) |

Abbreviations: NaSSA, noradrenergic and specific serotonergic antidepressant; SNRI, serotonin–norepinephrine reuptake inhibitor; SSRI, selective serotonin reuptake inhibitor; VOR, vortioxetine.

**TABLE S5 |** Comorbidities during the look-back period among patients with single prescriptions.

| **Comorbidities, n (%)** | **Single prescriptions** | | | | | |
| --- | --- | --- | --- | --- | --- | --- |
|  | **VOR  (n = 26 914)** | **SSRI  (n = 127 372)** | **SNRI  (n = 40 194)** | **NaSSA**  **(n = 25 847)** | **Other  (n = 36 623)** | **Overall (N = 256 950)** |
| Alcohol use disorders | 100 (0.4) | 422 (0.3) | 172 (0.4) | 172 (0.7) | 398  (1.1) | 1264 (0.5) |
| Other substance use disorders | 44 (0.2) | 262 (0.2) | 100 (0.2) | 64 (0.2) | 121 (0.3) | 591 (0.2) |
| Bipolar disorder | 813 (3.0) | 3123 (2.5) | 1130 (2.8) | 668 (2.6) | 1329 (3.6) | 7063 (2.7) |
| Depression | 16 548 (61.5) | 76 659 (60.2) | 24 855 (61.8) | 15 895 (61.5) | 22 443 (61.3) | 156 400 (60.9) |
| Anxiety disorders | 5537 (20.6) | 32 682 (25.7) | 6875 (17.1) | 4841 (18.7) | 6596 (18.0) | 56 531 (22.0) |
| Obsessive-compulsive disorder | 117 (0.4) | 1998 (1.6) | 95 (0.2) | 36 (0.1) | 112 (0.3) | 2358 (0.9) |
| PTSD | 53 (0.2) | 329 (0.3) | 48 (0.1) | 41 (0.2) | 72 (0.2) | 543 (0.2) |
| Adjustment disorders | 4010 (14.9) | 12 751 (10.0) | 3819 (9.5) | 3219 (12.5) | 3147 (8.6) | 26 946 (10.5) |
| Other stress reactions | 58 (0.2) | 297 (0.2) | 87 (0.2) | 83 (0.3) | 108 (0.3) | 633 (0.2) |
| Dissociative disorders | 236 (0.9) | 1303 (1.0) | 445 (1.1) | 263 (1.0) | 707 (1.9) | 2954 (1.1) |
| Somatoform disorders | 1639 (6.1) | 9579 (7.5) | 2698 (6.7) | 1879 (7.3) | 2542 (6.9) | 18 337 (7.1) |
| Sleep disorders | 11 351 (42.2) | 47 069 (37.0) | 16 228 (40.4) | 12 130 (46.9) | 17 180 (46.9) | 103 958 (40.5) |
| Personality disorders | 48 (0.2) | 292 (0.2) | 58 (0.1) | 54 (0.2) | 51 (0.1) | 503 (0.2) |
| Autism | 12 (< 0.1) | 87 (0.1) | 17 (< 0.1) | 12 (< 0.1) | 23 (0.1) | 151 (0.1) |
| Hyperactivity disorder | 640 (2.4) | 2515 (2.0) | 904 (2.2) | 319 (1.2) | 718 (2.0) | 5096 (2.0) |
| Dementia | 10 (< 0.1) | 78 (0.1) | 22 (0.1) | 31 (0.1) | 90 (0.2) | 231 (0.1) |
| Parkinson’s disease | 47 (0.2) | 306 (0.2) | 143 (0.4) | 80 (0.3) | 229 (0.6) | 805 (0.3) |
| Epilepsy | 385 (1.4) | 2083 (1.6) | 810 (2.0) | 497 (1.9) | 1501 (4.1) | 5276 (2.1) |
| Headaches | 3100 (11.5) | 15 812 (12.4) | 5659 (14.1) | 3471 (13.4) | 8840 (24.1) | 36 882 (14.4) |
| Autonomic nervous system disorders | 827 (3.1) | 4468 (3.5) | 1286 (3.2) | 839 (3.2) | 1104 (3.0) | 8524 (3.3) |
| Pain | 5062 (18.8) | 25 465 (20.0) | 11 657 (29.0) | 6241 (24.1) | 11 387 (31.1) | 59 812 (23.3) |
| Cancer | 2110 (7.8) | 12 157 (9.5) | 3953 (9.8) | 2907 (11.2) | 4872 (13.3) | 25 999 (10.1) |
| Diabetes mellitus | 1629 (6.1) | 7607 (6.0) | 3368 (8.4) | 2207 (8.5) | 3649 (10.0) | 18 460 (7.2) |
| Hypertensive disorders | 2563 (9.5) | 12 540 (9.8) | 5578 (13.9) | 3727 (14.4) | 5954 (16.3) | 30 362 (11.8) |
| Hyperlipidemia | 3079 (11.4) | 14 638 (11.5) | 6248 (15.5) | 4041 (15.6) | 6384 (17.4) | 34 390 (13.4) |
| Other noninfectious gastroenteritis and noninfectious colitis | 218 (0.8) | 1396 (1.1) | 488 (1.2) | 342 (1.3) | 565 (1.5) | 3009 (1.2) |
| Irritable bowel syndrome | 1021 (3.8) | 5390 (4.2) | 1750 (4.4) | 1163 (4.5) | 1828 (5.0) | 11 152 (4.3) |
| Nausea and vomiting | 2441 (9.1) | 10 957 (8.6) | 3456 (8.6) | 2099 (8.1) | 3328 (9.1) | 22 281 (8.7) |
| Hypothyroidism | 396 (1.5) | 2024 (1.6) | 664 (1.7) | 486 (1.9) | 727 (2.0) | 4297 (1.7) |
| Constipation | 1620 (6.0) | 9446 (7.4) | 3634 (9.0) | 2662 (10.3) | 4667 (12.7) | 22 029 (8.6) |
| Diarrhea | 6 (< 0.1) | 53 (< 0.1) | 15 (< 0.1) | 7 (< 0.1) | 17 (< 0.1) | 98 (< 0.1) |
| Somnolence | 11 (< 0.1) | 20 (< 0.1) | 11 (< 0.1) | 6 (< 0.1) | 6 (< 0.1) | 54 (< 0.1) |
| Premenstrual Syndrome | 120 (0.4) | 1242 (1.0) | 171 (0.4) | 86 (0.3) | 164 (0.4) | 1783 (0.7) |

Abbreviation: PTSD, post-traumatic stress disorder.

**TABLE S6 |** Concomitant medications during the look-back period among patients with single prescriptions.

| **Concomitant medications, n (%)** | **Single prescriptions** | | | | | |
| --- | --- | --- | --- | --- | --- | --- |
|  | **VOR  (n = 26 914)** | **SSRI  (n = 127 372)** | **SNRI  (n = 40 194)** | **NaSSA**  **(n = 25 847)** | **Other  (n = 36 623)** | **Overall  (N = 256 950)** |
| Anesthetics | 2669 (9.9) | 14 171 (11.1) | 6093 (15.2) | 3898 (15.1) | 6334 (17.3) | 33 165 (12.9) |
| Analgesics | 6875 (25.5) | 32 701 (25.7) | 12 876 (32.0) | 7325 (28.3) | 13 518 (36.9) | 73 295 (28.5) |
| Antiepileptic drugs | 651 (2.4) | 3292 (2.6) | 1343 (3.3) | 796 (3.1) | 2464 (6.7) | 8546 (3.3) |
| Antiparkinsonian | 99 (0.4) | 624 (0.5) | 211 (0.5) | 159 (0.6) | 305 (0.8) | 1398 (0.5) |
| Antipsychotics | 2886 (10.7) | 12 120 (9.5) | 4825 (12.0) | 3248 (12.6) | 4437 (12.1) | 27 516 (10.7) |
| Anxiolytics | 6217 (23.1) | 33 247 (26.1) | 9285 (23.1) | 6890 (26.7) | 9227 (25.2) | 64 866 (25.2) |
| Hypnotics and sedatives | 5990 (22.3) | 25 726 (20.2) | 9120 (22.7) | 8445 (32.7) | 12 840 (35.1) | 62 121 (24.2) |
| Antidepressants (excluding protocol-defined drugs) | 0 (0.0) | 0 (0.0) | 0 (0.0) | 0 (0.0) | 0 (0.0) | 0 (0.0) |
| Psychostimulants, ADHD medications and psychotropics | 392 (1.5) | 1628 (1.3) | 653 (1.6) | 228 (0.9) | 672 (1.8) | 3573 (1.4) |
| Antidementia drugs | 7 (< 0.1) | 35 (< 0.1) | 10 (< 0.1) | 17 (0.1) | 52 (0.1) | 121 (< 0.1) |
| Other nervous system medications | 903 (3.4) | 5175 (4.1) | 1506 (3.7) | 1105 (4.3) | 1703 (4.7) | 10 392 (4.0) |
| Antacids, bowel regulators, and ulcer medications | 7693 (28.6) | 38 366 (30.1) | 14 238 (35.4) | 9218 (35.7) | 14 385 (39.3) | 83 900 (32.7) |
| Drugs for functional gastrointestinal disorders | 4316 (16.0) | 22 150 (17.4) | 7393 (18.4) | 5657 (21.9) | 7883 (21.5) | 47 399 (18.4) |
| Antiemetics and antinauseants | 49 (0.2) | 279 (0.2) | 332 (0.8) | 286 (1.1) | 483 (1.3) | 1429 (0.6) |
| Laxatives | 1174 (4.4) | 6279 (4.9) | 2529 (6.3) | 1945 (7.5) | 3286 (9.0) | 15 213 (5.9) |
| Intestinal disease Preparations | 3330 (12.4) | 16 737 (13.1) | 5328 (13.3) | 3898 (15.1) | 5428 (14.8) | 34 721 (13.5) |
| Diabetes medications | 695 (2.6) | 2889 (2.3) | 1488 (3.7) | 912 (3.5) | 1740 (4.8) | 7724 (3.0) |
| Calcium channel blockers | 1245 (4.6) | 6254 (4.9) | 2873 (7.1) | 1976 (7.6) | 3175 (8.7) | 15 523 (6.0) |
| Renin angiotensin system agents | 1426 (5.3) | 6486 (5.1) | 3085 (7.7) | 2061 (8.0) | 3105 (8.5) | 16 163 (6.3) |
| Lipid control/atherosclerosis | 1831 (6.8) | 8522 (6.7) | 3832 (9.5) | 2503 (9.7) | 4014 (11.0) | 20 702 (8.1) |
| Anti-inflammatory and anti-rheumatic products | 6713 (24.9) | 31 665 (24.9) | 12 684 (31.6) | 7009 (27.1) | 12 289 (33.6) | 70 360 (27.4) |
| Topical anti-rheumatic drugs | 2859 (10.6) | 14 413 (11.3) | 6944 (17.3) | 3638 (14.1) | 6144 (16.8) | 33 998 (13.2) |
| Systemic corticosteroids | 2245 (8.3) | 11 896 (9.3) | 4995 (12.4) | 2705 (10.5) | 4973 (13.6) | 26 814 (10.4) |
| Thyroid therapeutics | 261 (1.0) | 1500 (1.2) | 453 (1.1) | 333 (1.3) | 504 (1.4) | 3051 (1.2) |
| Interferon | 0 (0.0) | 8 (< 0.1) | 6 (< 0.1) | 1 (< 0.1) | 3 (< 0.1) | 18 (< 0.1) |

Abbreviations: ADHD, attention-deficit/hyperactivity disorder.

**TABLE S7 |** Univariate regression analysis for the factors influencing the choice of vortioxetine as a first-line drug.

| **Explanatory variable** | **Odds ratio (95% CI)** |
| --- | --- |
| Sex (reference: male) |  |
| Female | 0.756 (0.737–0.775) |
| Age^a^ (reference: 20–60 years) |  |
| < 20 years | 0.902 (0.836–0.973) |
| > 60 years | 0.429 (0.393–0.467) |
| Comorbidities (reference: no) |  |
| Somnolence | 2.187 (1.128–4.242) |
| Adjustment disorders | 1.581 (1.525–1.639) |
| Hyperactivity disorder | 1.233 (1.134–1.341) |
| Bipolar disorder | 1.115 (1.036–1.201) |
| Sleep disorders | 1.082 (1.055–1.110) |
| Nausea and vomiting | 1.057 (1.011–1.104) |
| PTSD | 0.927 (0.698–1.230) |
| Autonomic nervous system disorders | 0.916 (0.851–0.985) |
| Anxiety disorders | 0.909 (0.882–0.938) |
| Personality disorders | 0.905 (0.672–1.219) |
| Hypothyroidism | 0.866 (0.780–0.961) |
| Other stress reactions | 0.862 (0.658–1.129) |
| Irritable bowel syndrome | 0.856 (0.802–0.914) |
| Somatoform disorders | 0.829 (0.786–0.873) |
| Hyperlipidemia | 0.820 (0.788–0.853) |
| Diabetes mellitus | 0.816 (0.774–0.860) |
| Hypertensive disorders | 0.766 (0.734–0.799) |
| Headaches | 0.756 (0.727–0.787) |
| Pain | 0.742 (0.718–0.766) |
| Dissociative disorders | 0.740 (0.648–0.846) |
| Autism | 0.738 (0.409–1.331) |
| Cancer | 0.734 (0.701–0.769) |
| Alcohol use disorders | 0.734 (0.598–0.900) |
| Other substance use disorders | 0.688 (0.506–0.935) |
| Epilepsy | 0.669 (0.603–0.743) |
| Other noninfectious gastroenteritis and noninfectious colitis | 0.666 (0.580–0.765) |
| Constipation | 0.658 (0.624–0.693) |
| Premenstrual syndrome | 0.615 (0.511–0.741) |
| Diarrhea | 0.561 (0.246–1.278) |
| Parkinson’s disease | 0.529 (0.394–0.711) |
| Obsessive-compulsive disorder | 0.444 (0.368–0.535) |
| Dementia | 0.387 (0.205–0.729) |
| Concomitant medications (reference: no) |  |
| Psychostimulants, ADHD medications, and psychotropics | 1.055 (0.949–1.173) |
| Antipsychotics | 1.002 (0.962–1.043) |
| Intestinal disease preparations | 0.894 (0.860–0.928) |
| Hypnotics and sedatives | 0.887 (0.861–0.914) |
| Anxiolytics | 0.878 (0.852–0.904) |
| Anti-inflammatory and anti-rheumatic products | 0.869 (0.844–0.894) |
| Analgesics | 0.845 (0.821–0.870) |
| Diabetes medications | 0.841 (0.777–0.910) |
| Drugs for functional gastrointestinal disorders | 0.829 (0.801–0.858) |
| Lipid control/atherosclerosis | 0.817 (0.777–0.859) |
| Renin angiotensin system agents | 0.817 (0.773–0.864) |
| Antacids, bowel regulators, and ulcer medications | 0.808 (0.786–0.831) |
| Other nervous system medications | 0.807 (0.753–0.865) |
| Thyroid therapeutics | 0.798 (0.702–0.906) |
| Systemic corticosteroids | 0.761 (0.728–0.796) |
| Topical anti-rheumatic drugs | 0.759 (0.729–0.791) |
| Calcium channel blockers | 0.733 (0.691–0.778) |
| Anesthetics | 0.721 (0.691–0.751) |
| Laxatives | 0.702 (0.661–0.746) |
| Antiepileptic drugs | 0.698 (0.644–0.757) |
| Antidementia drugs | 0.529 (0.247–1.132) |
| Antiemetics and antinauseants | 0.303 (0.228–0.403) |
| Interferon | < 0.001 (NA) |
| Antidepressants (excluding protocol-defined drugs) | NA |

Abbreviations: ADHD, attention-deficit/hyperactivity disorder; CI, confidence interval; NA, not available; PTSD, post-traumatic stress disorder.

^a^Age at the index date.

**TABLE S8 |** Univariate regression analysis for factors influencing treatment change of vortioxetine.

| **Explanatory variable** | **Odds ratio (95% CI)** |
| --- | --- |
| Sex (reference: male) |  |
| Female | 1.150 (1.067–1.239) |
| Age^a^ (reference: 20–60 years) |  |
| < 20 years | 1.107 (0.864–1.419) |
| > 60 years | 0.893 (0.686–1.161) |
| Comorbidities (reference: no) |  |
| Other substance use disorders | 1.909 (0.893–4.079) |
| Personality disorders | 1.685 (0.706–4.019) |
| Nausea and vomiting | 1.300 (1.150–1.468) |
| Other noninfectious gastroenteritis and noninfectious colitis | 1.229 (0.827–1.826) |
| Headaches | 1.228 (1.101–1.370) |
| Epilepsy | 1.222 (0.917–1.628) |
| Irritable bowel syndrome | 1.165 (0.970–1.400) |
| Dissociative disorders | 1.162 (0.808–1.672) |
| Sleep disorders | 1.150 (1.067–1.238) |
| Bipolar disorder | 1.150 (0.933–1.417) |
| Autonomic nervous system disorders | 1.147 (0.940–1.399) |
| Constipation | 1.134 (0.978–1.314) |
| Alcohol use disorders | 1.107 (0.611–2.006) |
| Hypothyroidism | 1.101 (0.815–1.488) |
| Premenstrual syndrome | 1.100 (0.649–1.864) |
| Pain | 1.054 (0.961–1.156) |
| Cancer | 1.054 (0.922–1.205) |
| Diabetes mellitus | 1.016 (0.879–1.173) |
| Hyperlipidemia | 0.968 (0.867–1.081) |
| Anxiety disorders | 0.957 (0.874–1.048) |
| Parkinson’s disease | 0.931 (0.372–2.332) |
| Hypertensive disorders | 0.929 (0.825–1.046) |
| Hyperactivity disorder | 0.899 (0.700–1.154) |
| Adjustment disorders | 0.897 (0.807–0.996) |
| Somatoform disorders | 0.889 (0.764–1.035) |
| PTSD | 0.841 (0.339–2.085) |
| Other stress reactions | 0.803 (0.325–1.981) |
| Somnolence | 0.736 (0.082–6.587) |
| Dementia | 0.590 (0.069–5.043) |
| Obsessive-compulsive disorder | 0.578 (0.303–1.104) |
| Autism | < 0.001 (NA) |
| Diarrhea | < 0.001 (NA) |
| Concomitant medications (reference: no) |  |
| Intestinal disease preparations | 1.427 (1.284–1.585) |
| Drugs for functional gastrointestinal disorders | 1.300 (1.183–1.429) |
| Other nervous system medications | 1.276 (1.062–1.534) |
| Antipsychotics | 1.206 (1.079–1.348) |
| Antacids, bowel regulators, and ulcer medications | 1.180 (1.090–1.278) |
| Anti-inflammatory and anti-rheumatic products | 1.170 (1.076–1.272) |
| Systemic corticosteroids | 1.135 (0.997–1.291) |
| Analgesics | 1.120 (1.031–1.217) |
| Laxatives | 1.108 (0.936–1.313) |
| Anesthetics | 1.091 (0.971–1.226) |
| Hypnotics and sedatives | 1.079 (0.991–1.175) |
| Antiepileptic drugs | 1.009 (0.800–1.273) |
| Anxiolytics | 0.996 (0.915–1.084) |
| Antidementia drugs | 0.981 (0.102–9.438) |
| Calcium channel blockers | 0.959 (0.814–1.129) |
| Thyroid therapeutics | 0.956 (0.662–1.381) |
| Topical anti-rheumatic drugs | 0.954 (0.846–1.075) |
| Lipid control/atherosclerosis | 0.898 (0.782–1.032) |
| Diabetes medications | 0.874 (0.700–1.091) |
| Renin angiotensin system agents | 0.823 (0.702–0.964) |
| Psychostimulants, ADHD medications and psychotropics | 0.788 (0.570–1.090) |
| Antiemetics and antinauseants | 0.701 (0.264–1.860) |
| Antidepressants (excluding protocol-defined drugs) | NA |
| Interferon | NA |

Abbreviations: ADHD, attention-deficit/hyperactivity disorder; CI, confidence interval; NA, not available; PTSD, post-traumatic stress disorder.

^a^Age at the index date.

**TABLE S9 |** Logistic regression analysis for factors influencing the treatment change of antidepressants.

| **First-line drug** | **Explanatory variable** | **Odds ratio (95% CI)**^a^ |
| --- | --- | --- |
| SSRI | Comorbidities (reference: no) |  |
|  | Parkinson’s disease | 1.674 (1.225–2.287) |
|  | Sleep disorders | 1.202 (1.158–1.248) |
|  | Nausea and vomiting | 1.083 (1.016–1.154) |
|  | Headaches | 1.068 (1.012–1.127) |
|  | Adjustment disorders | 0.936 (0.882–0.993) |
|  | Constipation | 0.899 (0.839–0.965) |
|  | Anxiety disorders | 0.884 (0.847–0.923) |
|  | Bipolar disorder | 0.876 (0.780–0.985) |
|  | Obsessive-compulsive disorder | 0.850 (0.742–0.974) |
|  | Premenstrual syndrome | 0.808 (0.661–0.988) |
|  | Dissociative disorders | 0.800 (0.669–0.957) |
|  | Hyperactivity disorder | 0.755 (0.657–0.867) |
|  | Dementia | 0.168 (0.040–0.698) |
|  | Concomitant medications (reference: no) |  |
|  | Antipsychotics | 1.178 (1.109–1.251) |
|  | Drugs for functional gastrointestinal disorders | 1.072 (1.016–1.131) |
|  | Intestinal disease preparations | 1.072 (1.015–1.133) |
|  | Anti-inflammatory and anti-rheumatic products | 1.068 (1.020–1.119) |
|  | Antacids, bowel regulators, and ulcer medications | 1.068 (1.019–1.120) |
|  | Anxiolytics | 0.911 (0.871–0.952) |
|  | Lipid control/atherosclerosis | 0.877 (0.818–0.939) |
| SNRI | Sex (reference: male) |  |
|  | Female | 1.149 (1.076–1.227) |
|  | Age^b^ (reference: 20–60 years) |  |
|  | < 20 years | 1.381 (1.109–1.719) |
|  | > 60 years | 0.695 (0.585–0.826) |
|  | Comorbidities (reference: no) |  |
|  | Sleep disorders | 1.196 (1.122–1.275) |
|  | Anxiety disorders | 1.100 (1.014–1.193) |
|  | Pain | 0.873 (0.809–0.941) |
|  | Constipation | 0.829 (0.734–0.937) |
|  | Concomitant medications (reference: no) |  |
|  | Intestinal disease preparations | 1.180 (1.072–1.300) |
|  | Antipsychotics | 1.161 (1.060–1.272) |
|  | Drugs for functional gastrointestinal disorders | 1.101 (1.009–1.202) |
|  | Antiepileptic drugs | 0.808 (0.676–0.966) |
|  | Lipid control/atherosclerosis | 0.807 (0.722–0.902) |
|  | Psychostimulants, ADHD medications, and psychotropics | 0.691 (0.531–0.899) |
|  | Antiemetics and antinauseants | 0.476 (0.286–0.791) |
| NaSSA | Sex (reference: male) |  |
|  | Female | 1.168 (1.083–1.259) |
|  | Age^b^ (reference: 20–60 years) |  |
|  | < 20 years | 1.039 (0.794–1.360) |
|  | > 60 years | 0.677 (0.574–0.799) |
|  | Comorbidities (reference: no) |  |
|  | Headaches | 1.132 (1.018–1.258) |
|  | Hypertensive disorders | 0.802 (0.719–0.895) |
|  | Bipolar disorder | 0.668 (0.517–0.862) |
|  | Concomitant medications (reference: no) |  |
|  | Drugs for functional gastrointestinal disorders | 1.168 (1.052–1.296) |
|  | Intestinal disease preparations | 1.166 (1.046–1.299) |
|  | Anxiolytics | 0.866 (0.796–0.942) |
|  | Anesthetics | 0.860 (0.766–0.966) |
|  | Antiepileptic drugs | 0.753 (0.599–0.945) |
|  | Antiemetics and antinauseants | 0.617 (0.396–0.960) |
| Other | Sex (reference: male) |  |
|  | Female | 0.900 (0.836–0.970) |
|  | Age^b^ (reference: 20–60 years) |  |
|  | < 20 years | 1.081 (0.841–1.389) |
|  | > 60 years | 0.682 (0.587–0.792) |
|  | Comorbidities (reference: no) |  |
|  | Somatoform disorders | 1.391 (1.221–1.584) |
|  | Anxiety disorders | 1.135 (1.036–1.243) |
|  | Sleep disorders | 1.110 (1.009–1.221) |
|  | Hyperlipidemia | 0.798 (0.722–0.883) |
|  | Dissociative disorders | 0.727 (0.542–0.976) |
|  | Headaches | 0.633 (0.578–0.694) |
|  | Hyperactivity disorder | 0.553 (0.384–0.796) |
|  | Alcohol use disorders | 0.449 (0.298–0.678) |
|  | Concomitant medications (reference: no) |  |
|  | Intestinal disease preparations | 1.300 (1.175–1.438) |
|  | Anesthetics | 1.166 (1.053–1.291) |
|  | Antipsychotics | 1.142 (1.028–1.269) |
|  | Hypnotics and sedatives | 0.747 (0.677–0.825) |
|  | Psychostimulants, ADHD medications, and psychotropics | 0.612 (0.422–0.887) |
|  | Antiepileptic drugs | 0.571 (0.486–0.670) |
| Overall | Age^b^ (reference: 20–60 years) |  |
|  | < 20 years | 1.091 (1.012–1.176) |
|  | > 60 years | 0.893 (0.834–0.957) |
|  | Comorbidities (reference: no) |  |
|  | Parkinson’s disease | 1.259 (1.021–1.553) |
|  | Sleep disorders | 1.189 (1.158–1.220) |
|  | Anxiety disorders | 0.928 (0.900–0.958) |
|  | Hypertensive disorders | 0.928 (0.890–0.967) |
|  | Constipation | 0.914 (0.871–0.958) |
|  | Bipolar disorder | 0.909 (0.840–0.983) |
|  | Dissociative disorders | 0.872 (0.769–0.988) |
|  | Hyperactivity disorder | 0.846 (0.751–0.952) |
|  | Premenstrual syndrome | 0.831 (0.710–0.974) |
|  | Obsessive-compulsive disorder | 0.784 (0.693–0.888) |
|  | Autism | 0.447 (0.229–0.872) |
|  | Dementia | 0.363 (0.194–0.681) |
|  | Concomitant medications (reference: no) |  |
|  | Antipsychotics | 1.175 (1.129–1.223) |
|  | Intestinal disease preparations | 1.150 (1.107–1.195) |
|  | Drugs for functional gastrointestinal disorders | 1.106 (1.068–1.146) |
|  | Antacids, bowel regulators, and ulcer medications | 1.053 (1.019–1.088) |
|  | Anti-inflammatory and anti-rheumatic products | 1.049 (1.017–1.083) |
|  | Anxiolytics | 0.925 (0.897–0.954) |
|  | Lipid control/atherosclerosis | 0.874 (0.833–0.918) |
|  | Antiepileptic drugs | 0.829 (0.768–0.895) |
|  | Psychostimulants, ADHD medications, and psychotropics | 0.784 (0.680–0.904) |
|  | Antiemetics and antinauseants | 0.773 (0.630–0.947) |

Abbreviations: ADHD, attention-deficit/hyperactivity disorder; CI, confidence interval; NaSSA, noradrenergic and specific serotonergic antidepressant; SNRI, serotonin–norepinephrine reuptake inhibitor; SSRI, selective serotonin reuptake inhibitor.

^a^All factors were statistically significant at p < 0.05 in stepwise multivariate logistic regression. ^b^Age at the index date.
